# Long Distance Linkage Disequilibrium and Limited Hybridization Suggest Cryptic Speciation in Atlantic Cod

**DOI:** 10.1371/journal.pone.0106380

**Published:** 2014-09-26

**Authors:** Ian R. Bradbury, Sharen Bowman, Tudor Borza, Paul V. R. Snelgrove, Jeffrey A. Hutchings, Paul R. Berg, Naiara Rodríguez-Ezpeleta, Jackie Lighten, Daniel E. Ruzzante, Christopher Taggart, Paul Bentzen

**Affiliations:** 1 Department of Fisheries and Oceans, St. John’s, Newfoundland, Canada; 2 Marine Gene Probe Laboratory, Department of Biology, Dalhousie University, Halifax, Nova Scotia; 3 The Atlantic Genome Centre, Halifax, Nova Scotia, Canada; 4 Ocean Sciences Center, Memorial University of Newfoundland, St. John’s, Newfoundland, Canada; 5 Centre for Ecological and Evolutionary Synthesis, Department of Biology, University of Oslo, Oslo, Norway; 6 AZTI-Tecnalia, Marine Research Division, Txatxarramendi ugartea z/g, Sukarrieta, Spain; 7 Department of Oceanography, Dalhousie University, Halifax, Nova Scotia, Canada; Nanjing Forestry University, China

## Abstract

Hybrid zones provide unprecedented opportunity for the study of the evolution of reproductive isolation, and the extent of hybridization across individuals and genomes can illuminate the degree of isolation. We examine patterns of interchromosomal linkage disequilibrium (ILD) and the presence of hybridization in Atlantic cod, *Gadus morhua*, in previously identified hybrid zones in the North Atlantic. Here, previously identified clinal loci were mapped to the cod genome with most (∼70%) occurring in or associated with (<5 kb) coding regions representing a diverse array of possible functions and pathways. Despite the observation that clinal loci were distributed across three linkage groups, elevated ILD was observed among all groups of clinal loci and strongest in comparisons involving a region of low recombination along linkage group 7. Evidence of ILD supports a hypothesis of divergence hitchhiking transitioning to genome hitchhiking consistent with reproductive isolation. This hypothesis is supported by Bayesian characterization of hybrid classes present and we find evidence of common F1 hybrids in several regions consistent with frequent interbreeding, yet little evidence of F2 or backcrossed individuals. This work suggests that significant barriers to hybridization and introgression exist among these co-occurring groups of cod either through strong selection against hybrid individuals, or genetic incompatibility and intrinsic barriers to hybridization. In either case, the presence of strong clinal trends, and little gene flow despite extensive hybridization supports a hypothesis of reproductive isolation and cryptic speciation in Atlantic cod. Further work is required to test the degree and nature of reproductive isolation in this species.

## Introduction

Understanding the complex contributions of ecological factors and genomic architecture to the formation of reproductive isolation is central to an understanding of speciation [1]–[3]. Evidence is accumulating that isolation and speciation can occur despite gene flow where divergent selection exists for different habitats or environments [4]–[6]. Study of the early stages of speciation where hybridization is common can reveal genome features involved, as well as the dominant isolating mechanisms (e.g., [5], [7], [8]). These processes are particularly apparent in hybrid zones and among ecological species where admixture commonly occurs and selection or barriers to hybridization maintain reproductive isolation [9]–[11]. Accordingly, genome wide examinations of hybrid individuals may provide unprecedented insight into the genomic architecture associated with speciation, identify sources and targets of selection [2], [3], [12], and clarify management and conservation objectives [13].

Recent studies continue to highlight the importance of genomic architecture to speciation and adaptation [14]–[16] and that patterns of linkage disequilibrium (LD) are more complex than previously thought (e.g., [17], [18]). Better descriptions of both the genomic distribution of divergence and LD are necessary if architecture underlying adaptive divergence, and ultimately, the mechanisms underlying speciation are to be resolved [12], [19]. How important “islands of genomic divergence” are to adaptation and speciation remains unclear, but it seems likely they play an important role during the early stages of speciation [2], [12]. Speciation or isolation with gene flow is hypothesized to involve a progression of increasing isolation leading from selection at a few isolated genomic regions to genome wide divergence [1]. During this process, islands of genomic divergence may grow in size via divergence hitchhiking. Both the magnitude and size of these islands of adaptive divergence will depend on the genomic architecture and strength of selection [2], [6]. Genomic island size has been reported to be between a few to hundreds of kb [20], and islands may be smaller and more isolated when gene flow is high [12], [17], [21], [22]. As such, these genome wide patterns of divergence (i.e. frequency and size of islands of divergence, and LD among islands) may directly reflect the degree of reproductive isolation present between intraspecific groups.

Atlantic cod, *Gadus morhua*, is a commercially exploited demersal marine fish found throughout the North Atlantic characterized by high dispersal potential, large fecundities, and large population sizes [23]. Given the wide range of environments occupied, cod are likely exposed to a large range of ocean climates from temperate to high latitudes. We recently used SNP-based genome scans in Atlantic cod (*G. morhua*) in conjunction with linkage mapping to demonstrate the utility of genomic islands of adaptive divergence [16] in management and conservation efforts. These initial genomic explorations for the presence of islands of adaptive divergence identified a subset of gene-associated polymorphisms for which allele frequencies displayed parallel latitudinal clines (here designated as N (north) and S (south) types) in otherwise genetically distinct populations on both sides of the Atlantic and tested positive for signatures of selection [24]. SNPs associated with these types were non-randomly distributed across the genome and mapped to three linkage groups. Recent studies and extensive sampling have confirmed the presence of two distinct forms of two distinct groups of Atlantic cod [16] and that subsets of these SNPs are commonly identified as outliers, often associated with temperature [25]–[27]. The geographic co-occurrence of these forms and presence of distinct hybrid zones allows the examination of mechanisms involved in both genomic and reproductive isolation.

The goal of the present paper is to examine the nature of the trends observed previously (e.g., [16], [24]) and in particular to explore patterns of interchromosomal linkage disequilibrium (ILD) and the presence of hybridization between wild Atlantic cod N and S types in the previously identified hybrid zone in the northwest Atlantic. First, we identify possible functional associations of clinal SNPs through alignment with the recently available Atlantic cod genome, and an exploration of SNP-gene associations. Second, we explore ILD among SNPs in three different linkage groups previously associated with clinal structure. Finally, we examine the degree of hybridization among these two latitudinal cod types using a Bayesian approach to assign individuals to discrete hybrid classes. We build on previous studies, which examined loci displaying evidence of environmentally associated selection in parallel on either side of the Atlantic in a subset of the loci (n = 40) and populations (n = 14) used in this study [24], and the application of these loci for fisheries management and conservation [16], [28]. Here, we delve further into the mechanisms driving these patterns and present evidence of previously unknown cryptic speciation and divergence in a common, heavily exploited marine fish. The implications for the management of Atlantic cod as well as the potential for cryptic diversity in other well studied and exploited marine organisms (e.g., [13]) suggest significant gaps in our current understanding perhaps best addressed with population genomic approaches.

## Materials and Methods

### Sample characteristics

We re-analyzed data on 23 Atlantic cod populations all included in Bradbury *et al*. (2013) and explored the presence of LD and hybridization in the wild. These data represent cod sampled (*N* = 466) from throughout the North Atlantic in 1996–2007, and approximately 20 individuals were sampled from each location (Figure S1 in File S1). Specific details regarding all samples and locations were published elsewhere [24], [29]–[31]. This study primarily dealt with existing DNA or tissue samples and did not involve the handling of live specimens. This study used tissue samples from fish collected during routine Government of Canada sampling of fish stocks under the supervision of the departmental animal care committee. Fish collected as part of assessment activities are exempt from animal care protocols as they are being collected under our regulatory mandate for establishing abundance estimates. This exemption is found in section 4.1.2.2 of the Canadian Council on Animal Care’s Guidelines on the use of fish in research, teaching and testing. As all fish collected in this manner are deceased prior to handling, all tissue samples were collected post mortem. We isolated DNA from these samples from ethanol-preserved fin clips using a modification of a previously published glass milk procedure [32]. These samples were genotyped for 1536 SNPs (GenBankdbSNP under accession numbers ss131570222-ss131571915). Details on SNP development and genotyping were provided elsewhere [31], [33]. SNPs were genotyped using the Illumina Golden Gate platform at the McGill Innovation Center. Linkage mapping information is available for most of these SNPs based on three families, including parents and F1 offspring [34].

### Annotation and SNP alignment

Here we focus on linkage groups containing clinal (latitude) SNP outliers (i.e. LG2, LG7. LG12). Of SNPs previously identified as outliers, most could not be annotated using BLAST [24]. We attempted to identify associations with adjacent genes, through alignment with the recently published cod genome to identify potential ontological/functional relationships. These outlier SNPs and 80–90 bp of flanking sequence were mapped to the cod genome scaffold [35] using default parameters in BLAT [36] implemented in the Ensemble Genome Browser (http://www.ensembl.org/Gadus_morhua/Info/Index). We accepted only E values >1.0e^−40^ with sequence similarity >96% as a hit to the correct position in the Atlantic cod genome. In the instance that a SNP did not map to a protein coding region of the genome, the distance (bases) to the nearest flanking genes on either side were calculated. SNPs within annotated genes and SNPs within a 5 Kb distance from an annotated gene were given a HGNC (Human Genome Nomenclature Committee) ID. We also used these annotations from the cod genome scaffold to obtain gene ontology and functionality information from the Uniprot (www.uniprot.org), or GeneCards (www.genecards.org) databases.

### Linkage and divergence

In previous work, outlier tests and spatial analysis [16], [24] identified a subset of these loci (n = 53, Table S1 in File S1) that displayed elevated divergence and signatures indicative of directional selection, and most were associated with latitudinal clines. These SNPs were distributed across three linkage groups. Here, we evaluated the presence of long distance ILD among all loci on the linkage groups containing outliers. LD was quantified with D’ estimated using TASSEL [37]. D’ represents the D statistic which measures the non-random association among alleles at two loci, standardized by its maximum possible value, and ranges from 0 representing no association to 1 representing complete linkage. The three linkage groups previously shown to contain outliers associated with latitudinal clinal structure were assessed for ILD among loci. D’ was measured among SNPs along each combination of these groups. Similarly *F*_ST_ was calculated for each locus again in these three linkage groups using ARLEQUIN [38]. Both measures of D’ and *F*_ST_ were examined in conjunction with map position using the linkage map [34].

### Bayesian Tests for Hybridization

Two Bayesian approaches were used to infer the presence and frequency of various hybrid classes in the wild using only the outlier loci. Bayesian clustering was performed using STRUCTURE v.2.2.4 [39] to estimate admixture coefficients for each individual. This approach assumes Hardy-Weinberg Equilibrium (HWE) and linkage equilibria among loci, introduces population structure, and assigns populations that are not in linkage equilibrium using a Markov chain Monte Carlo (MCMC) algorithm to estimate the number of populations (K). The algorithm was run 5 times for K = 2 to ensure convergence of values, and with a burn-in of 100 000 repetitions, 300 000 repetitions after burn-in. STRUCTURE was also run with 1 million repetitions to ensure 300 000 were sufficient. All STRUCTURE results were amalgamated among replicates using CLUMPP 1.1.2 [40] and summarized graphically using DISTRUCT 1.1 [41].

In addition to STRUCTURE, NEWHYBRIDS [42] was to used examine hybridization and the presence of specific hybrid classes. NEWBYBRIDS uses a Gibbs sampler and Markov chain Monte Carlo to estimate the posterior probability that genetically sampled individuals fall into each of a total of six set of hybrid or parentage categories (pure N, pure S; F1 hybrids; F2 hybrids; and both backcrosses). NEWHYBRIDS was run with a burn-in of 100 000 iterations followed by 250,000 iterations and individuals belonging to a category with probability >90% were considered correctly assigned.

To evaluate the ability of each approach to correctly identify the various hybrid classes, individuals of each class were simulated using HYBRIDLAB 1.0 [43]. All six parentage groups (N-type, S-type, F1 hybrids, F2 hybrids and reciprocal backcrosses) were simulated under random mating using 50 N-type and S-type individuals. To identify these 50 pure individuals of each group, the STRUCTURE results were filtered for individuals with admixture coefficients >0.90 and <0.10. HYBRIDLAB was then used to simulate 50 individuals of each hybrid class. Known N- and S-type as well as simulated hybrids were then used to evaluate assignment accuracy using both STRUCTURE and NEWHYBRIDS, implementing similar parameters and methods for each as described above.

## Results

### Clines and SNP annotation

**Figure 1:**
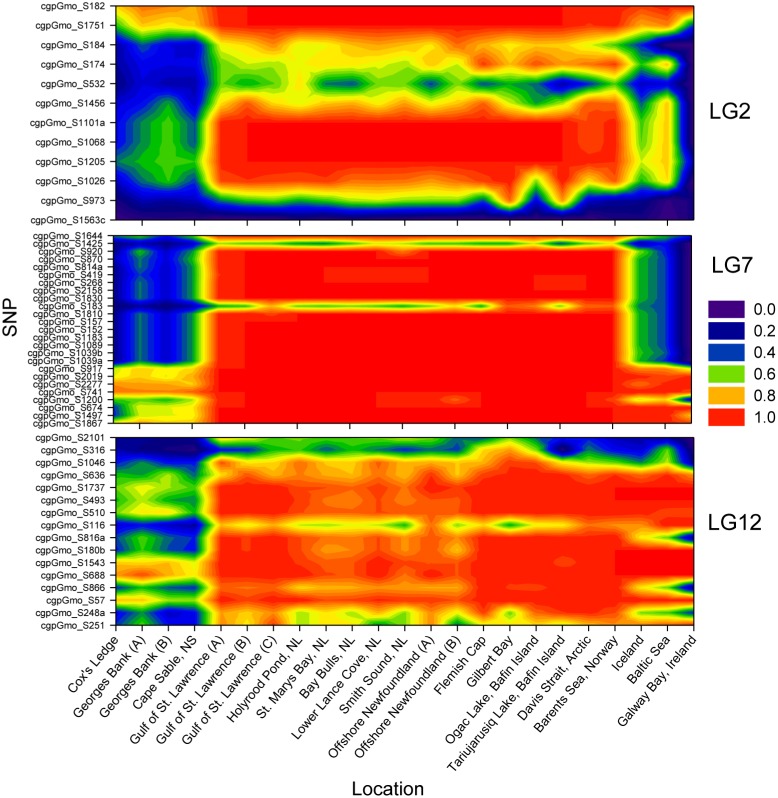
Clinal trends in allele frequency in Atlantic cod range-wide. Allele frequencies for outlier loci in linkage groups LG2, LG7 and LG12 from 23 locations. See Bradbury et al. [16] for specific results of outlier tests. See Figure S1 in File S1 for sample locations.

Outlier loci (see [16] for outlier test details) in linkage groups characterized by the presence of clinal loci [24] were re-evaluated using data on new populations. LG2 contained 12 outliers, the majority of which displayed parallel clines in both the east and west Atlantic (Figure 1). In LG7, most of 25 identified outliers displayed clear clinal trends in allele frequency in both the East and West Atlantic. Similarly LG12 displayed clinal trends in allele frequency in most outliers present. Of these three, LG7 displayed the most consistent patterns in allele frequency across locations and loci with very little variation among most individual and locus comparisons. Overall, trends among most outliers in all three linkage groups were very similar and contained 73% of all outliers identified (see [16]).

To identify genes containing or associated with (<5 kb) these SNPs, SNP sequences were mapped to cod genome scaffold [44]. Of the 53 outlier SNPs contained on these three linkage groups, 38 SNPs or ∼70% were associated with known coding regions. Of these, nine mapped within gene regions, and 30 mapped within 5 kb of a known coding region in gene scaffolds (see Table S1 in File S1). Only two SNPs (cgpGMO_S182 and cgpGMO_S184) were adjacent to the same gene, MYLPF (i.e. myosin light chain, phosphorylatable, fast skeletal muscle) supporting the outlier status of this gene involved in muscle activity. In general, the identified SNP-associated genes represent a diverse array of function and possible physiological pathways. For example, cgpGmo_S917 is associated with AQP11 (Aquaporin 11) which is thought to be related to water transport (and associated with Na/K ATPase activity) across cell membranes and hence could play a key role in adaptation to different water conditions etc. Similarly, cgpGmo_S510 is associated with FASL (Fas ligand (TNF superfamily, member 6), which has been associated with signalling pathways and the immune response. Refer to Table S1 in File S1 for individual SNP results of alignment and annotation.

### Interchromosomal Linkage Disequilibrium

**Figure 2:**
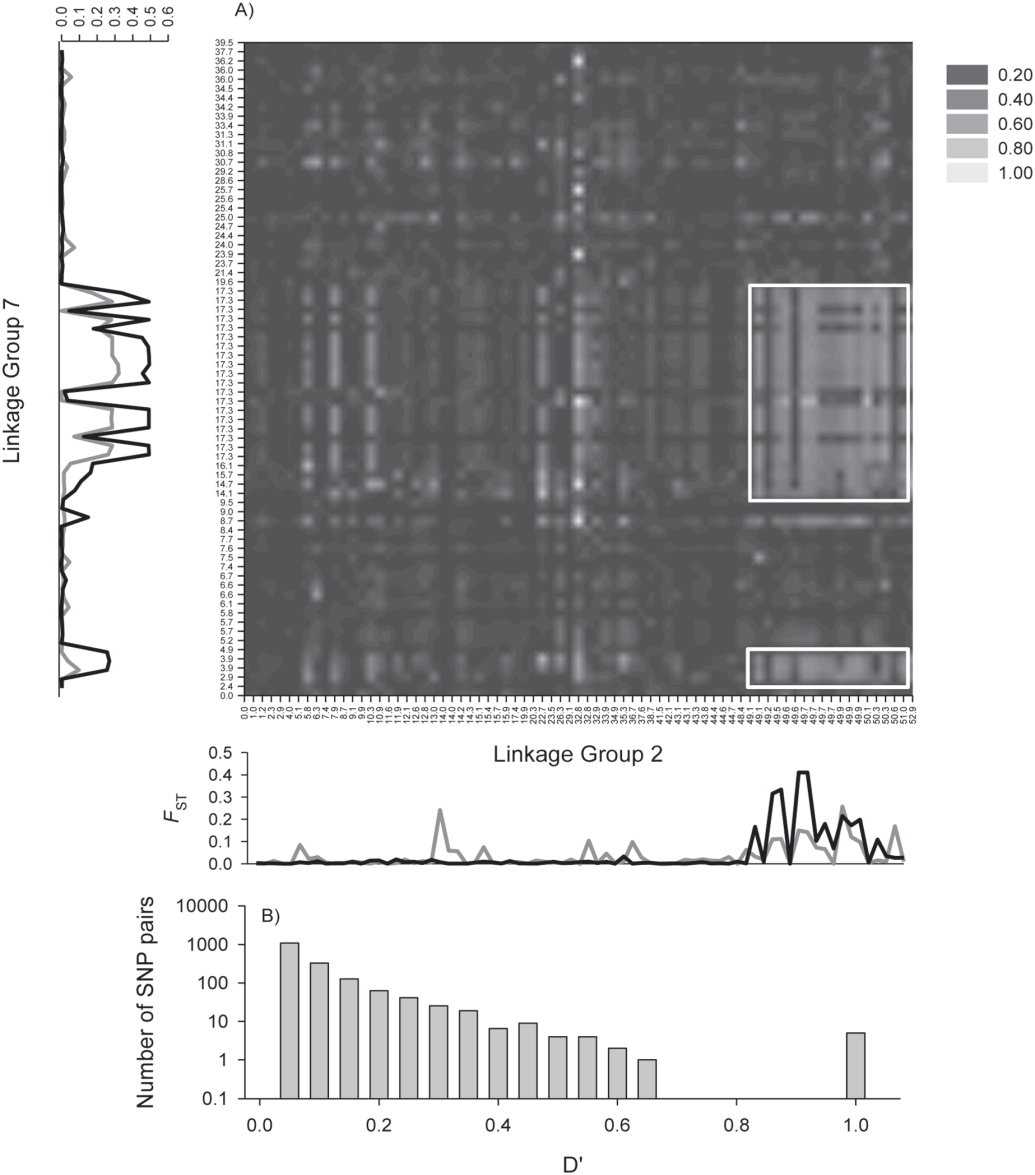
Interchromosomal linkage disequilibrium between LG2 and LG7. (A) Heat map of interchromosomal linkage disequilibrium between LG2 and LG7, axis labels represent linkage map positions (see [34]), note that several SNPs share a common map location. Plots of population differentiation (*F*_ST_) on the left and the bottom compare divergence between N and S types, in both the west (black line) and east Atlantic (grey line). Two main regions of ILD between previously identified adaptive genomic regions are highlighted by white rectangles. (B) Histogram of D’ values for all interchromosomal pairs of SNPs.

**Figure 3:**
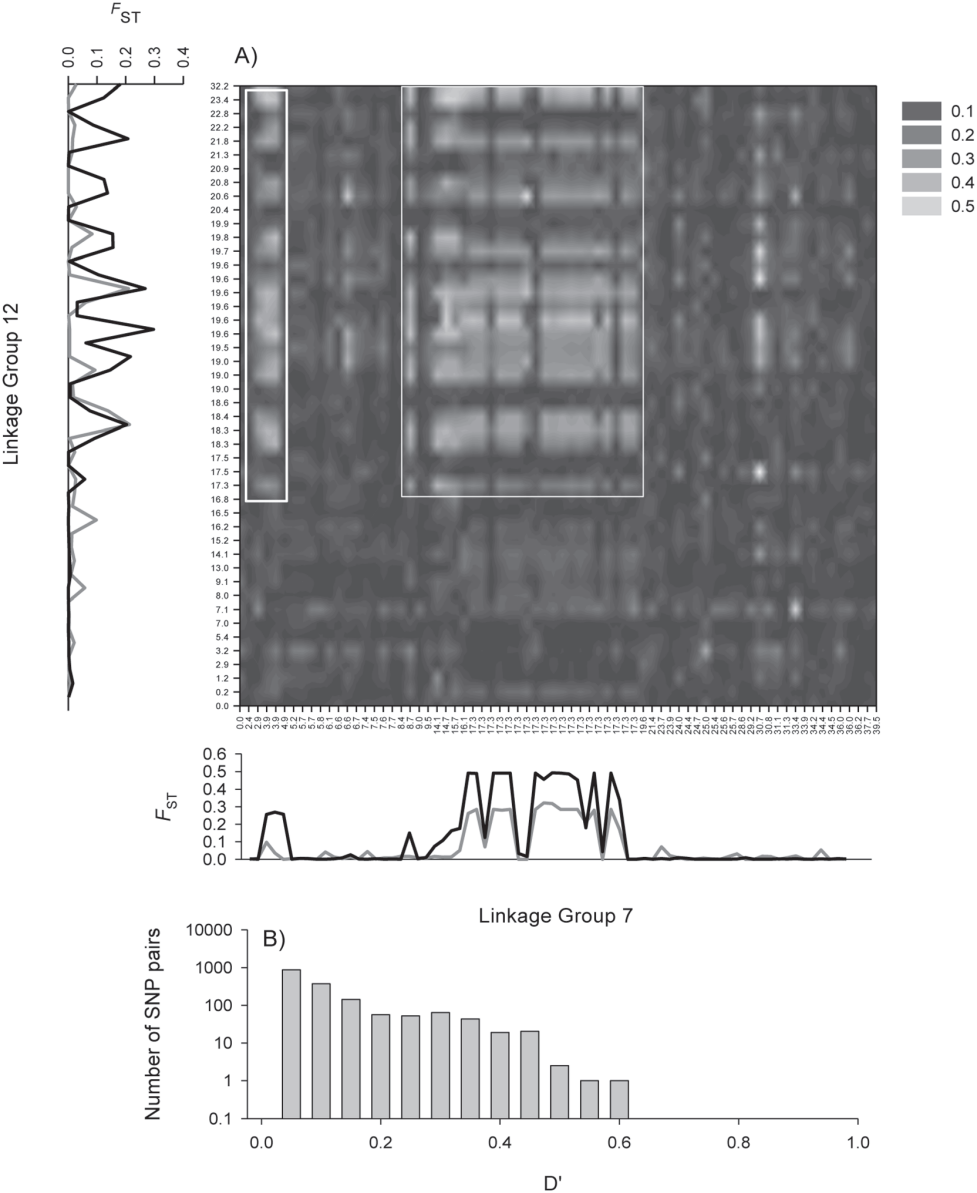
Interchromosomal linkage disequilibrium between LG7 and LG12. (A) Heat map of interchromosomal linkage disequilibrium between LG7 and LG12, axis labels represent linkage map positions (see [34]), note that several SNPs share a common map location. Plots of population differentiation (*F*_ST_) on the left and the bottom compare divergence between N and S types, in both the west (black line) and east Atlantic (grey line). Two main regions of ILD between previously identified adaptive genomic regions are highlighted by white rectangles. (B) Histogram of D’ values for all interchromosomal pairs of SNPs.

**Figure 4:**
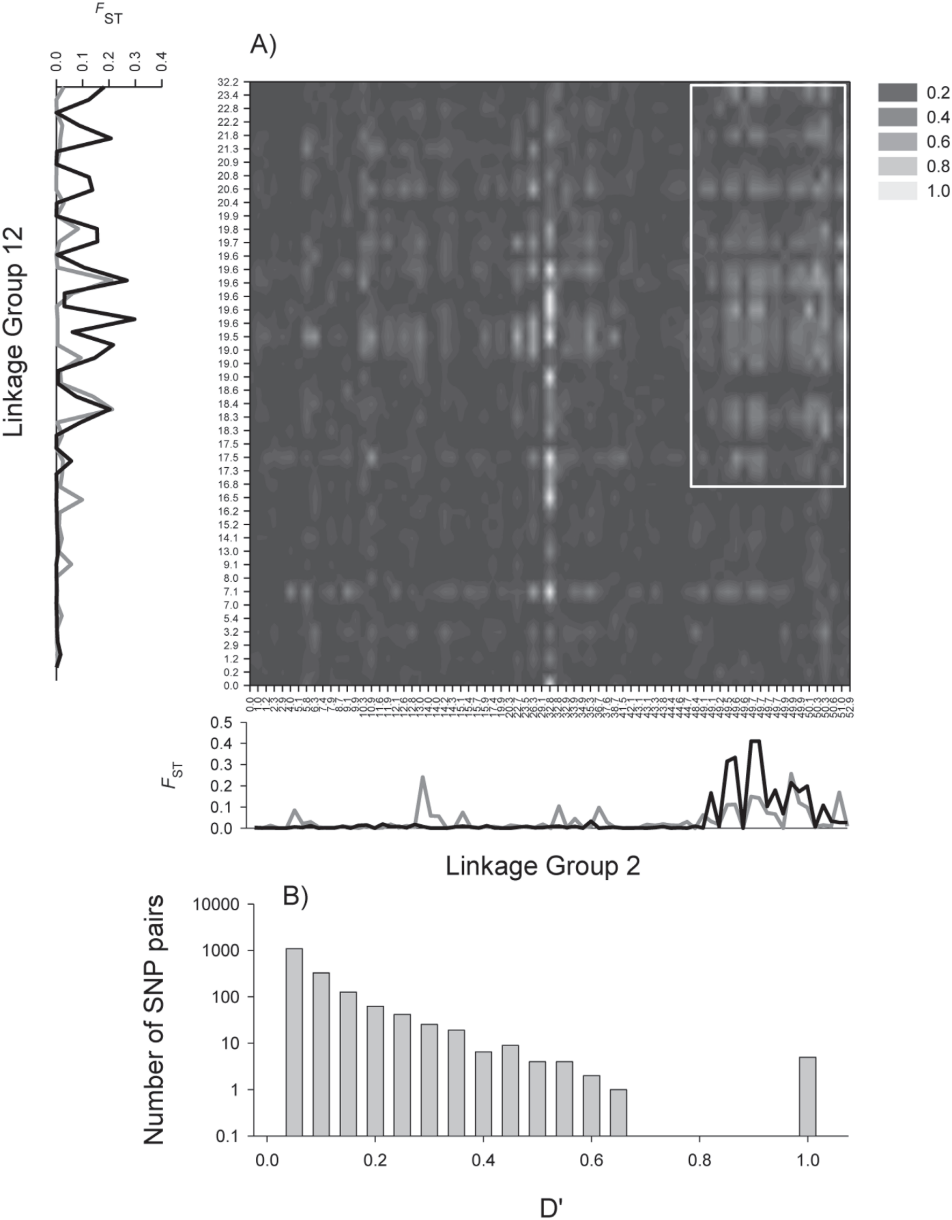
Interchromosomal linkage disequilibrium between LG2 and LG12. (A) Heat map of interchromosomal linkage disequilibrium between LG2 and LG12, axis labels represent linkage map positions (see [34]), note that several SNPs share a common map location. Plots of population differentiation (*F*_ST_) on the left and the bottom compare divergence between N and S types, in both the west (black line) and east Atlantic (grey line). Two main regions of ILD between previously identified adaptive genomic regions are highlighted by white rectangles. (B) Histogram of D’ values for all interchromosomal pairs of SNPs.

Significant ILD was observed among all three linkage groups and in general regions of significant LD were associated with elevated divergence (i.e. *F*_ST_). The largest region of consistent high divergence and D’ was near the middle of LG7. This region displayed some of the highest LD values and most consistent in comparison to the other LG’s. A uniform block of approximately 4 cM near the centre of LG7 was strongly associated with an approximately 3 cM section of LG2 (Figure 2). This region was also associated with high *F*_ST_ values between the two forms at the outlier loci on either side of the North Atlantic (Figure 2, Figure S2 in File S1). For these comparisons, D’ values ranged from 0 to 1, but many loci had values >0.5 (Figure 2b). This same section of LG7 was also associated with LG12 as indicated by elevated D’ values along a 15 cM section (Figure 3). Again this region was also associated with high *F*_ST_ values between the two forms at the outlier loci on either side of the North Atlantic (Figure 3a and Figure S2 in File S1) and D’ values ranged from 0 to 0.6 with 10’s of loci >0.5 (Figure 3b). LG12 also displayed lower overall divergence at outlier loci than did LG2 or LG7. Regions of LG2 and LG12 which had been associated with LG7 and elevated divergence among the two forms, displayed some evidence of ILD with each other (Figure 4a, b).

#### Hybridization analysis

**Figure 5:**
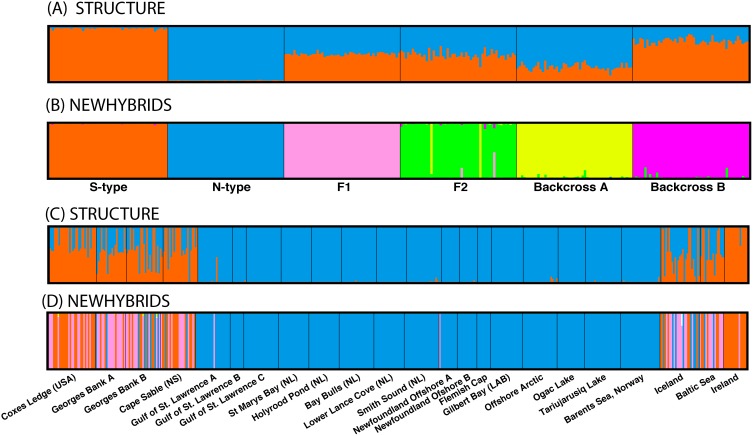
Bayesian assignment of simulated hybrid classes using (A) Structure with K = 2, and (B) New Hybrids with six possible hybrid classes of individuals (N type, S type, F1, F2, Backcross A, and Backcross B). Individuals simulated using HYBRIDLAB. Bayesian identification of hybrid classes of individuals present in wild Atlantic cod samples using (C) Structure, and (D) New Hybrids.

The power to detect hybrid individuals and specific hybrid classes was evaluated using simulated hybrid individuals (see Methods). Using STRUCTURE, pure individuals were readily identified and distinguished from hybrids. However, F1, F2, and backcrossed individuals could not easily be differentiated as both were characterized by intermediate admixture coefficients (Figure 5a). In contrast, NEWHYBRIDs correctly identified on average 99% of all individuals across all hybrid classes (Figure 5b). Only F2 individuals were not correctly identified with 100% accuracy, with success dropping to 96%. This drop is due to two F2 individuals being incorrectly identified as backcrossed individuals (Figure 5b). Given the high success rate observed for the identification of at least some pure or hybrid groups, both analyses were conducted on field collected individuals to quantify the degree of hybridization and presence of each hybrid class.

**Figure 6:**
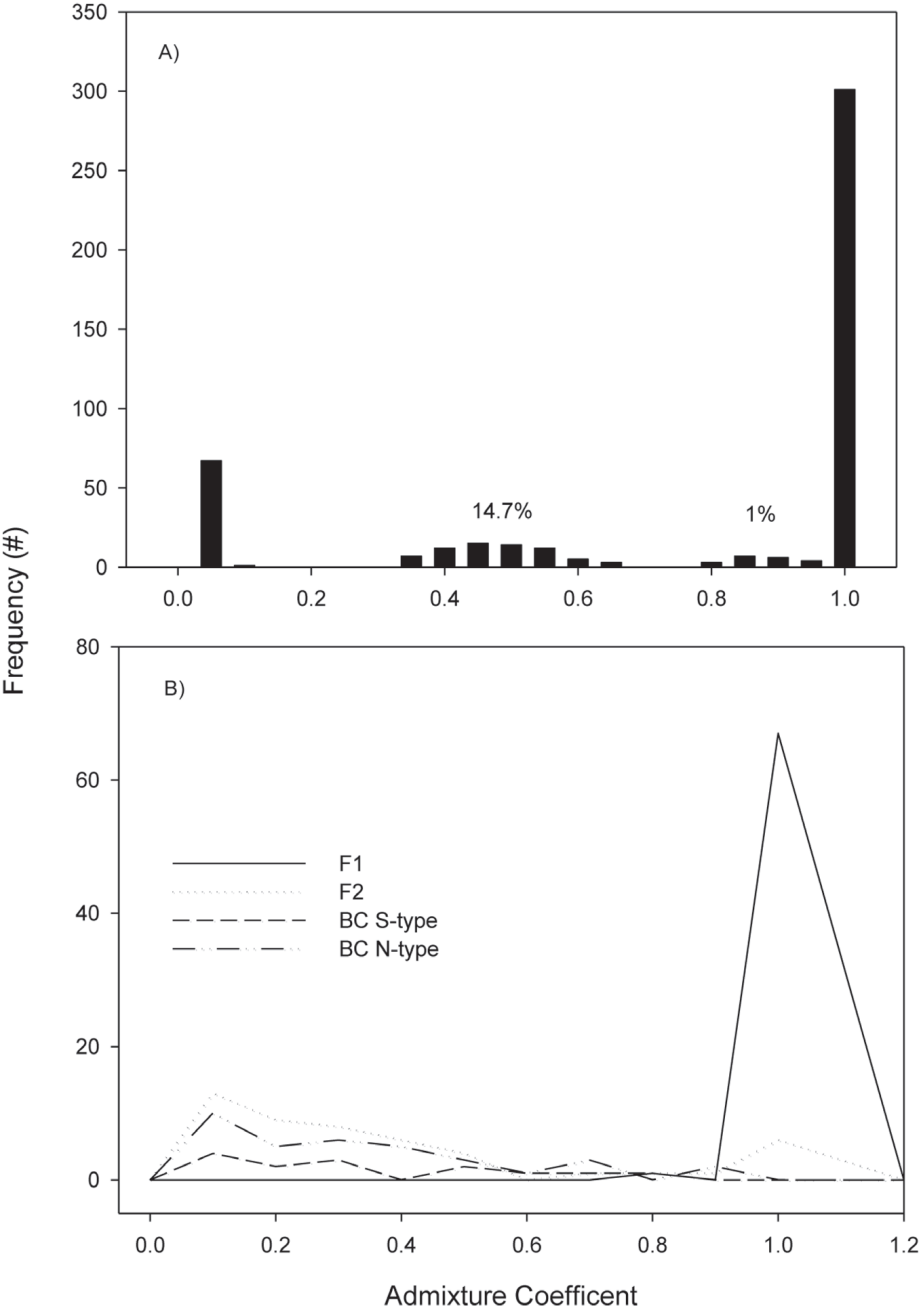
Frequency distributions of admixture coefficients from analysis of wild caught Atlantic cod using both (A) STRUCTURE and (B) NEWHYBRIDS.

In general on both sides of the Atlantic, northern samples (Norway to Gulf of St. Lawrence) were characterized by pure N-type, with S-type and hybrid individuals dominating to the south (Figure 5). On both sides of the Atlantic, the frequency of hybrid individuals declined to the north, a trend visible in both STRUCTURE and NEWHYBRIDS analyses. The exception seems to be the sample from Ireland, which was almost entirely pure S-type, except for one individual which was identified as an F1 (Figure 5). The STRUCTURE analysis indicated significant admixture in southern populations but the presence of exact hybrid classes was difficult to discern (Figure 5). In comparison, NEWHYBRIDS identified almost all admixed individuals as F1 with little evidence of F2 and no evidence of backcrossed individuals (Figure 6). In some samples, such as one from Georges Bank, the proportion of F1s was high (>50%) (Figure 5). Frequency plots of admixture coefficients from both analyses indicate the presence of admixed individuals overall was about 15% and comprised of almost entirely F1 individuals (Figure 6).

## Discussion

Hybridization along hybrid zones or between ecological species can provide rare opportunity for the study of the evolution of reproductive isolation (e.g., [3], [10], [45]). The degree of hybridization evident across genomes and the frequency of occurrence of hybrid classes can yield insight into the ecological and genomic processes associated with speciation [1], [12], [46]. The levels of linkage disequilibrium (LD) among outlier genes observed here and the lack of extensive gene flow are consistent with a hypothesis of two sympatric forms of Atlantic cod (N and S types) and support the hypothesis that these genomic regions are associated with reductions in hybrid survival and reproductive isolation. The presence of significant ILD between sympatric forms (e.g., [17], [18]) has been reported elsewhere and is consistent with the presence of genome hitchhiking, but the observation that the neutral markers displayed no association with these groups [24] also suggests that its influence has been limited. Moreover the observation that genomic regions associated with these two forms represent a diverse array of possible functions and the involvement of multiple physiological pathways is consistent with the influence of a very broad selective agent such as temperature, as has been suggested previously [24]. Our results challenge current management paradigms in this species which consider both groups a single management unit in the southern portions of the range. Further study is needed to examine the geographic distributions, ecological and physiological attributes, and reproductive interactions of these two groups, and further resolve the genomic details of the linkage groups and ILD patterns identified here. Similar observations of surprisingly high levels of biocomplexity in Atlantic cod have also been made at the range margin in the waters off Greenland [27] and the eastern Atlantic [26] suggesting this may be a common phenomenon in this species. It is worth noting that patterns observed here seem independent of the genomic differences reported among migratory and stationary cod populations in Norway [26], [47] and Iceland [48].

The parallel nature of environmental gradients in the North Atlantic provide replicated environmental and latitudinal gradients and a model for the identification of genes associated with ecological influences [49]. This work extends an earlier study identifying the clinal nature of the outlier loci associated with the N- and S-types in this species on either side of the North Atlantic [24] by extending the geographic coverage considerably through re-analysis of samples described in Bradbury et al. [16]. With additional sampling, steep clines in allele frequency at outlier loci are consistently identified along the Scotian shelf in the west and around Iceland or southern Norway in the east. Interestingly, although most outliers in the three linkage groups examined displayed parallel clines, >50% of the loci in LG12 displayed clines only in the west and were fixed in the east. Similar latitudinal clines have been reported in other species in the Atlantic [50] as well as in the Pacific (e.g., [51]). Previously we identified temperature associations with allele frequency at 40 of these outliers supporting a hypothesis of temperature associated selection [24]. An alternate hypothesis for the formation of these clines is that these are ”tension” zones formed by secondary contact of differentially adapted forms that have become trapped along environmental or ecological gradients [52]. Our observation of parallel clines requires this “trapping” of tension zones to have occurred independently on either side of the Atlantic, and as non-outliers show no evidence of clinal structure, this explanation seems less likely. Although at present the nature of these clines and whether they represent trapped tension zones or parallel adaptive evolution remains unknown, in the context of this study, the key point of interest is that the two forms (N and S type) appear genetically distinct and appear to resist introgression despite significant interbreeding where they co-occur.

Understanding the nature of these clinal loci such as whether or not they implicate similar physiological pathways or functions is critical to the interpretation of these clines. Previous attempts to explore the gene associations of the key SNPs that distinguish the N and S types using BLAST had very limited success, producing annotations of only a few sequences [24], [31], [33]. Here, taking advantage of the recently published cod genome [44], the majority of these SNPs have been located in genes or associated with genes, allowing us to explore the potential functional importance of these SNPs. Most of the SNPs associated with these N and S types are clustered in discrete regions of the genome [16], the question of how independent these types are is reasonable. The observation that all but two SNPs were associated with different genes, supports the involvement of a suite of genes in the observed clines rather than the linkage of most SNPs to a few clinal genes. These associated genes implicate a range of physiological pathways and functions with no obvious trend. This observation is consistent with the hypothesis of either a broad acting selective agent targeting numerous physiological functions (i.e. temperature), or perhaps longstanding genomic divergence and incompatibility among N and S forms. However, a hypothesis of longstanding genomic divergence does not seem compatible with observations of little to no divergence at the majority of SNPs examined. The lack of a clear and consistent link to function across many of the SNPs contrasts the outcomes of genome scans in other marine fishes, where outliers across environmental gradients are associated with genes of obvious functional importance. For example, Lamichhaney et al. [53] identify several outliers in Baltic herring populations with clear relevance to the strong salinity gradients present in the Baltic. Also, Limborg et al. [54] also identified environmentally corrected outliers in transcriptome derived SNPs in the northeast Atlantic. Or similarly, in Atlantic cod in the eastern Atlantic, Hemmer-Hansen et al. [26] identified a genomic island associated with migratory phenotype in coastal cod which included the Pantophysin gene.

The study raises the question of how the non-random association of genes on three different chromosomes can exist despite apparent high rates of interbreeding in some locations. The evidence of extensive LD among these outliers and islands of divergence on different chromosomes is consistent with either a lack of interbreeding, the suppression of recombination, or low hybrid fitness. A lack of successful interbreeding could explain the association among islands of divergence, but the apparent common occurrence of F1 individuals suggests this is not the case. However, as F2 or backcrossed individuals were rare, it is difficult to rule out intrinsic barriers to gene flow. In theory, transmission ratio distortion (e.g., [55]) could partly explain the trends in LD observed. However this was not readily visible in mapping crosses [34] and seems unlikely. Inter-chromosome translocations could potentially play a role and should be investigated, although it seems unlikely that this could easily explain the observed LD among three chromosomes. Hohenlohe et al. [17] report significant LD among adaptive genomic regions on two different chromosomes, in threespine stickleback in marine and freshwater habitats. They concluded that population structuring in conjunction with additive and epistatic selection provided the most parsimonious explanation for ILD across these stickleback genomes. Such an explanation seems most likely here as well, though will require further study. Similar trends in ILD have been reported both in *Drosophila* [18] and sticklebacks [17] and associated with reproductive isolation or ecological speciation. Also observations of ILD have been made in situations where populations are structured either through selective breeding or strong selection such as strains of the cultivated tomato [56] or in genes associated with drug resistance in *Plasmodium falciparum* [57].

The absence of most hybrid classes supports a hypothesis of reproductive isolation and limited gene flow, although several possible explanations exist for this apparent lack of hybrid classes in southern locations where the N and S forms co-occur. First, it is possible that this analysis lacked sufficient power to detect some hybrid classes. However, the simulation and analysis of hybrid classes supports the hypothesis that these individuals would have been correctly identified if present. A second possible explanation is that the presence of Bateson-Dobzhansky-Muller incompatibilities among islands of divergence may explain the observed linkage among chromosomes and the lack of some hybrid classes in the wild. The Bateson–Dobzhansky–Muller model predicts that postzygotic isolation evolves due to the accumulation of incompatible epistatic interactions, but few studies have quantified the relationship between genetic architecture and patterns of reproductive divergence [58]. Simulations of clines associated with BDM incompatibilities indicate that BDM epistasis with interacting locus pairs on different chromosomes resulted in the greatest long distance genomic autocorrelation in cline parameters [10]. This is largely consistent with our observations here. Finally, it is also possible that gynogenesis may play a role and explain the relatively high numbers of F1s observed, though further work is required to explore this possibility.

In addition to the lack of some hybrid classes, the high proportion of individuals classified as F1’s (>50%) in some samples was noteworthy. The number of individuals classified as F1’s was highest at the mid latitudes examined (i.e. Georges Bank) and declined to the south in the east and west, with only a single F1 individual being present in the sample from Ireland. Despite the high proportion of F1’s in some samples, the overall proportion among all samples was low (∼15%) suggesting that some samples or regions may have been biased towards hybridization or the collection of hybrids (e.g., [59]). Admittedly it remains unclear to what degree these linked genomic islands are a product of selection and speciation (“speciation island” hypothesis) or due to an absence of gene flow and the clustering of ancestral regions of divergence in regions of low recombination (“incidental island” hypothesis) [60]. Which scenario is more likely will require further genomic and experimental work.

Regardless of which scenario is valid in this case, observation here of genetically discrete N- and S-types despite interbreeding suggests reproductive isolation and an evolutionarily significant division. There is also some evidence that the forms possess divergent phenotypes (e.g., [61], [62]) and occupy different habitats (e.g., [24]). Accordingly it seems reasonable to posit that these forms represent cryptic or ecological species as has been suggested elsewhere [26]. Given the low levels of divergence across most of the genome among these forms it is reasonable to hypothesize that the ILD observed is associated with the a transition from divergence hitchhiking to genome hitchhiking in this species. A similar situation has been documented in *Anopheles* mosquitoes where two sympatric forms have been described [63] associated with islands of divergence on three chromosomes [18]. In this case, as here, identifying the role selection plays in maintaining these islands of divergence remains a challenge [18], [60]. From a conservation or management perspective, the parapatric occurrence of these discrete forms pose clear challenges for the conservation of diversity in this exploited species in southern locations as has been suggested for northern locations [27]. Ultimately, a better understanding of the spatial and temporal distribution of N- and S-types will be needed if this diversity is to be reflected in the current management framework for this species.

## Summary

Disentangling the contributions of ecological factors and genomic architecture to the formation of reproductive isolation is central to an understanding of speciation [1]–[3]. The distribution and degree of genomic divergence is expected to reflect the magnitude of reproductive isolation present among forms, and in theory can be used to evaluate where on the speciation continuum intraspecific forms reside, yet distinguishing islands of divergence due to selection and speciation from islands resulting from low regions of recombination remains a challenge. The results presented here provide convincing evidence that multiple clinal SNPs distributed across three linkage groups were associated with each other and reductions in the occurrence of hybrid classes in the wild. This work supports the hypothesis that in these sympatric N- and S-types of Atlantic cod, divergence has transitioned from divergence hitchhiking to genome hitchhiking. The results reveal cryptic divergence, possibly associated with ecological speciation in Atlantic cod on both sides of the North Atlantic. The implications of such unrecognized diversity in this extremely well studied marine fish are broad ranging, from challenging fisheries management and the conservation practices in Atlantic cod, to a broader recognition of increased biocomplexity in marine populations. Ultimately, the nature of these forms, the degree of reproductive isolation, and presence of extensive hybridization will require further field and lab study.

## Supporting Information

File S1 **Supporting Information file that includes Table S1, Figure S1, and Figure S2.** Table S1. SNP associated genes and map position from SNP sequence alignment to cod genome using BLAT. See methods for details. Figure S1. Map of sample locations for Atlantic cod tissue samples distributed throughout the North Atlantic. Figure S2. *F*_ST_ between N and S types from either side of the North Atlantic (yellow – west, red – east) with linkage map distance across (A) LG2, (B) LG7 and (C) LG12.
(PDF)
