# Supplementary material for: Long Distance Linkage Disequilibrium and Limited Hybridization Suggest Cryptic Speciation in Atlantic Cod
Source: PLoS One. 2014 Sep 26;9(9):e106380. doi: 10.1371/journal.pone.0106380 (PMC4178228; doi:10.1371/journal.pone.0106380)
Supplement: File S1 — Supporting Information file that includes Table S1, Figure S1, and Figure S2. Table S1. SNP associated genes and map position from SNP sequence alignment to cod genome using BLAT. See methods for details. Figure S1. Map of sample locations for Atlantic cod tissue samples distributed throughout the North Atlantic. Figure S2. F ST between N and S types from either side of the North Atlantic (yellow – west, red – east) with linkage map distance across (A) LG2, (B) LG7 and (C) LG12. (PDF) [file pone.0106380.s001.pdf]

Supplementary Information

for

# Long distance linkage disequilibrium and limited hybridization along parallel clines suggest ecological speciation in Atlantic cod

THE PDF INCLUDES:
TABLE S1
FIGURE S1-S2

**TABLE S1.** SNP associated genes and map position from SNP sequence alignment to cod genome using BLAT. See methods for details.

| SNP | LG | Position | Distance | Associated Gene |
|---|---|---|---|---|
| **cgpGmo_S973** | CGPIA2 | 56.189 | 0 | ribosomal protein L3 (65609-72352) |
| **cgpGmo_S1026** | CGPIA2 | 56.661 | 0 | solute carrier family 25, member 39 (73892-79384) |
| **cgpGmo_S1205** | CGPIA2 | 56.739 | 2676 | LIM domain containing 2 (185209-189564) |
| **cgpGmo_S1456** | CGPIA2 | 56.835 | 27 | KDEL (Lys-Asp-Glu-Leu) endoplasmic reticulum protein retention receptor 2 (26787-29049) |
| **cgpGmo_S1101a** | CGPIA2 | 56.835 | 2348 | sodium channel, voltage-gated, type IV, alpha subunit (1 of 2) 5271-30123) |
| **cgpGmo_S1068** | CGPIA2 | 56.835 | 979 | DDB1 and CUL4 associated factor 7 (5657-8696) |
| **cgpGmo_S532** | CGPIA2 | 56.835 | 410 | solute carrier family 35, member B1 (107497-111201) |
| **cgpGmo_S174** | CGPIA2 | 57.04 | 1341 | upstream binding transcription factor, RNA polymerase I (144721-152430) |
| **cgpGmo_S184** | CGPIA2 | 57.04 | 1771 | myosin light chain, phosphorylatable, fast skeletal muscle (193329-200381) |
| **cgpGmo_S1751** | CGPIA2 | 57.296 | 0 | acid-sensing (proton-gated) ion channel 2 (intronic) 41-106798) |
| **cgpGmo_S182** | CGPIA2 | 57.574 | 411 | myosin light chain, phosphorylatable, fast skeletal muscle (193329-200381) |
| **cgpGmo_S1867** | CGPIA7 | 6.436 | 1913 | pancreatic progenitor cell differentiation and proliferation factor homolog (zebrafish) (4924-6833) |
| **cgpGmo_S1497** | CGPIA7 | 7.43 | 343 | MIS18 kinetochore protein homolog A (S. pombe) (189927-195832) |
| **cgpGmo_S1200** | CGPIA7 | 9.272 | 136 | complement component 1, q subcomponent binding protein (1555907-1558956) |
| **cgpGmo_S741** | CGPIA7 | 15.235 | 3657 | transmembrane protein 248 (192964-199991) |
| **cgpGmo_S2019** | CGPIA7 | 17.391 | 3779 | solute carrier family 25 (mitochondrial carrier, brain), member 14 (4402-10474) |
| **cgpGmo_S917** | CGPIA7 | 18.267 | 517 | aquaporin 11 (161395-163638) |
| **cgpGmo_S268** | CGPIA7 | 19.147 | 0 | Cbl proto-oncogene, E3 ubiquitin protein ligase B (intronic) (56250-351047) |
| **cgpGmo_S419** | CGPIA7 | 19.147 | 917 | phosphatidylinositol binding clathrin assembly protein (4120-20931) |
| **cgpGmo_S870** | CGPIA7 | 19.147 | 0 | kaptin (actin binding protein) (72874-76785) |
| **cgpGmo_S157** | CGPIA7 | 19.147 | 226 | RNA exonuclease 2 homolog (S. cerevisiae) (33740-36263) |
| **cgpGmo_S2158** | CGPIA7 | 19.147 | 21 | EF-hand domain family, member A1 (272530-276927) |
| **cgpGmo_S920** | CGPIA7 | 19.147 | 1097 | acyl-CoA synthetase long-chain family member 6 (7285-33373) |

| | | | | |
|---|---|---|---|---|
| **cgpGmo_S183** | CGPIA7 | 19.147 | 1057 | BTG family, member 3 (527184-528326) |
| **cgpGmo_S1830** | CGPIA7 | 19.147 | 558 | aspartoacylase (298502-303228) |
| **cgpGmo_S251** | CGPIA12 | 14.796 | 0 | peroxiredoxin 6 (148413-153299) |
| **cgpGmo_S248a** | CGPIA12 | 15.582 | 3364 | solute carrier family 9, subfamily A (NHE7, cation proton antiporter 7), member 7 (515656-536260) |
| **cgpGmo_S57** | CGPIA12 | 16.59 | 0 | transmembrane and coiled-coil domains 1 (14892-17453) |
| **cgpGmo_S688** | CGPIA12 | 17.687 | 2789 | defective in cullin neddylation 1, domain containing 4 (S. cerevisiae) (193734-198924) |
| **cgpGmo_S816a** | CGPIA12 | 18.327 | 0 | tissue specific transplantation antigen P35B (31516-36175) |
| **cgpGmo_S180b** | CGPIA12 | 18.327 | 1336 | serine/arginine-rich splicing factor 11 (315493-322567) |
| **cgpGmo_S116** | CGPIA12 | 18.552 | 590 | ribosome production factor 1 homolog (S. cerevisiae) (1259357-1262063) |
| **cgpGmo_S510** | CGPIA12 | 18.726 | 41 | Fas ligand (TNF superfamily, member 6) (25301-27404) |
| **cgpGmo_S493** | CGPIA12 | 18.952 | 84 | TBC1 domain family, member 7 (170053-173134) |
| **cgpGmo_S1737** | CGPIA12 | 19.69 | 1273 | phosphatidic acid phosphatase type 2 domain containing 2 (153815-157086) |
| **cgpGmo_S636** | CGPIA12 | 19.814 | 2823 | transforming growth factor, beta receptor III (3172-59004) |
| **cgpGmo_S1046** | CGPIA12 | 20.994 | 0 | LIM domain containing preferred translocation partner in lipoma (intronic) (27660-154978) |
| **cgpGmo_S2101** | CGPIA12 | 34.413 | 1414 | ArfGAP with coiled-coil, ankyrin repeat and PH domains 2 (235598-290359) |

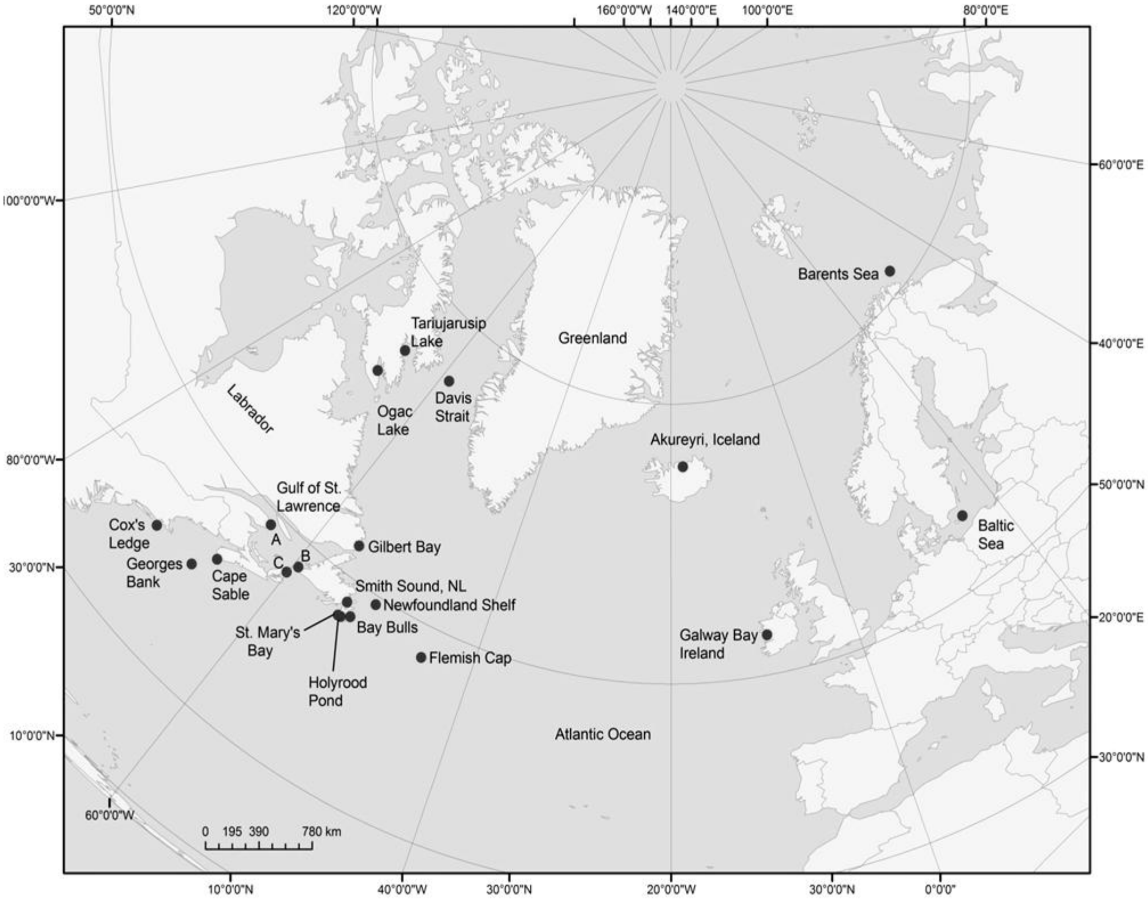


Figure S1. Map of sample locations for Atlantic cod tissue samples distributed throughout the North Atlantic.

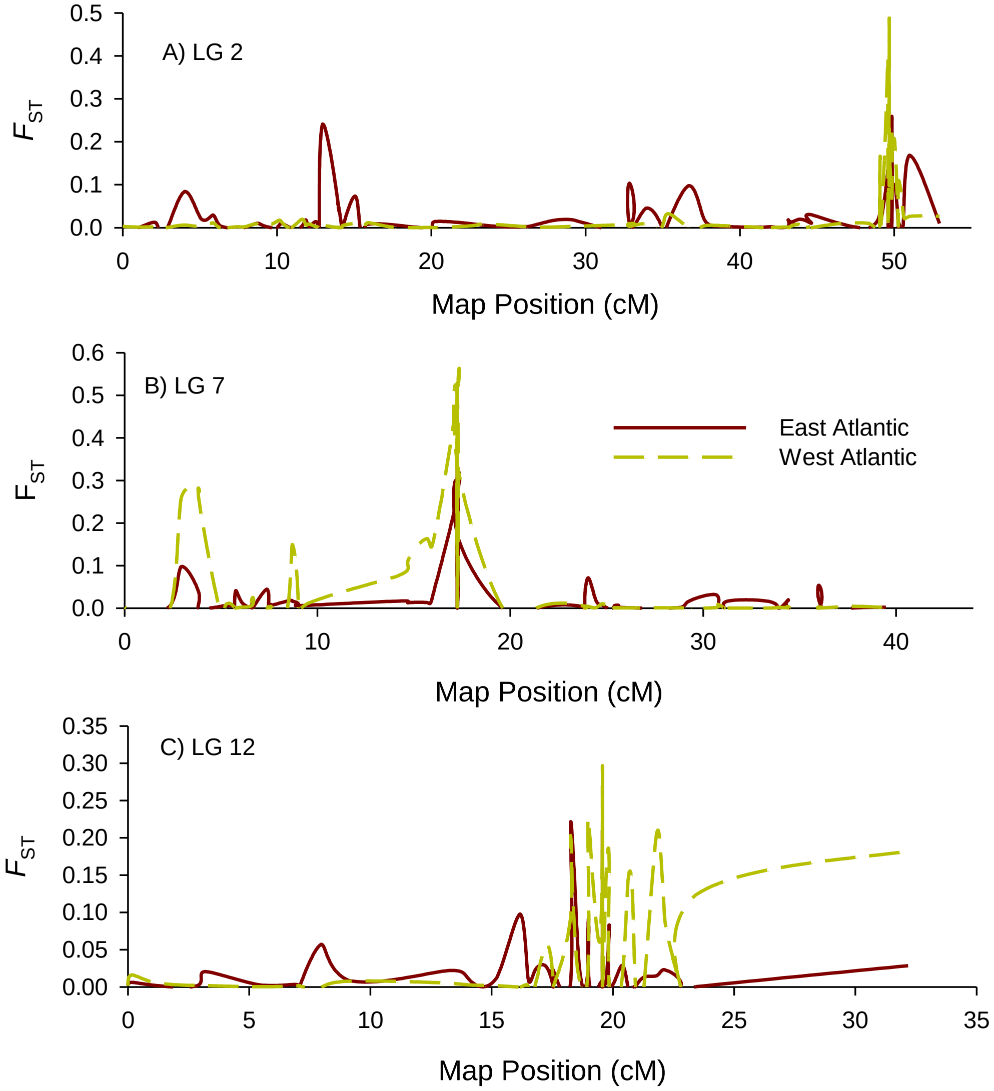


Figure S2. $F_{ST}$ between N and S types from either side of the North Atlantic (yellow – west, red – east) with linkage map distance across (A) LG2, (B) LG7 and (C) LG12.
